# Effectiveness of oral corticosteroids (prednisolone) in sensorineural hearing loss post COVID-19

**DOI:** 10.1186/s43163-022-00347-2

**Published:** 2022-12-14

**Authors:** Wessam Mostafa Essawy

**Affiliations:** Audio-vestibular Unit, Department of Otorhinolaryngology, Faculty of Medicine, Tanta University, Tanta, Egypt

**Keywords:** Sudden sensorineural hearing loss (SSNHL), COVID-19 infection, Corticosteroid therapy (methylprednisolone)

## Abstract

**Background:**

Numerous infections can result in neurological symptoms, including anosmia, facial paralysis, and abrupt sensorineural hearing loss (SSNHL). During the earlier SARS pandemic, coronaviruses were linked to a loss of smell and taste due to brain damage.

**Purpose:**

To clinically detect corticosteroid treatment effectiveness in SSNHL post-COVID-19 infection and to detect the factors that affect the prognosis for these patients for better diagnosis and earlier management.

**Subjects and method:**

Subjects included 20 subjects diagnosed by PCR as COVID-19 virus positive, complaining of sudden onset hearing loss post viral infection in different durations. All subjects had basic audiological evaluation done pre-treatment and repeated after 1 week, 2 weeks, and 1 month after treatment with methylprednisolone 21-acetate tablets.

**Results:**

Onset of hearing loss post-COVID infection ranged from 1 to 3 months. As regards the improvement recognized with treatment course, thirteen patients (65%) of all twenty patients showed complete improvement at 1 month after starting treatment, and seven patients (35%) showed no improvement even after 1 month.

**Conclusion:**

SSNHL has been widely recognized in the context of COVID-19 to date. Early corticosteroid therapy could help in the recovery of hearing, especially if the beginning of therapy was early in the first 2 weeks.

## Background

Multiple viruses can produce neurological symptoms, including anosmia, facial paralysis, and abrupt sensorineural hearing loss (SSNHL) [1–3].. Due to brain damage, infection with coronaviruses was associated with loss of smell and taste during the preceding pandemic of SARS [4, 5].

In Wuhan, China, severe acute respiratory syndrome coronavirus 2 (SARS-CoV-2) was discovered as the causal agent of coronavirus disease (COVID-19), which resulted in pneumonia and respiratory failure that led to death and was subsequently labeled a pandemic. It was revealed to be a coronavirus that is transmitted by droplets or direct contact with human airway epithelial cells [6].

The definition of SSNHL is sensorineural hearing loss of at least 30 dB at three consecutive frequencies within 72 h. In the pathophysiology of SSNHL due to viral infections, three mechanisms have been proposed: neuritis resulting from viral involvement of the cochlear nerves, cochleitis resulting from viral involvement of the cochlea and perilymphatic tissues, and the stress response resulting from the cross-reaction of inner ear antigens with viral antigens [7].

However, other alternative hypotheses have been put forth. First, viral infection has been frequently linked to SSNHL. In the past, SSNHL patients’ viral loads were verified using serological techniques, including PCR and immunoglobulin detection. The second hypothesized that the etiology of SSNHL is vascular dysfunction. Cochlear ischemia and hearing loss might theoretically be caused by cardiovascular risk factors, virus-induced hypercoagulability, and inflammatory edema [8].

Corticosteroids are essential to treat SSNHL [9]. Due to the possibility of worsening the disease and delaying viral clearance, the risks and benefits of using corticosteroids to treat viral infections remain controversial. By analyzing the presence of SARS-CoV-2 in individuals with SSNHL symptoms and using a number of therapy methods, it may be possible to prevent these undesirable effects [10].

Hearing recovery results in SSNHL have been linked to many variables. This list contains the age, severity, and kind of hearing impairment. Those with more severe hearing loss are more likely to have a poor prognosis [11, 12]. A flat or high-frequency slope audiogram is connected with a poor prognosis, but a low-frequency or mid-frequency hearing loss configuration is related to a greater likelihood of recovery [13]. The association between age and hearing recovery rates and threshold rises is consistent and unfavorable in terms of demographic factors [11, 12, 14].

To date, SSNHL has been well-recognized in relation to COVID-19. Anosmia induced by neuropathology associated with COVID-19 has been observed; however, the relationship between COVID-19 and sensorineural hearing loss is poorly understood. In otolaryngology, ongoing research focuses on the most effective steroid therapy for sensorineural hearing loss. This sparked the idea for this study, which sought to determine the clinical efficacy of corticosteroid treatment in SSNHL patients following COVID-19 infection, as well as the factors that influence prognosis in these patients for better diagnosis and earlier management.

### Subjects and method

Subjects in this study included one study group. It consists of 20 subjects diagnosed by PCR as COVID-19 virus positive, complaining of sudden onset hearing loss post viral infection in different durations.

## Methods

All participants in this study were subjected to the following:

1. Full audiological history including age, sex, ear affected, the onset of hearing loss post-COVID infection, the presence or absence of tinnitus and vertigo, duration between hearing loss and starting treatment, and general diseases (hypertension, DM, hepatic or renal affection). Also, the complete history of COVID infection, treatment, and hospitalization was taken for clinical correlation.
2. Otological examination
3. Basic audiological evaluation, including pure tone audiometry, immittancemetry, and speech audiometry. This evaluation was done pre-treatment and repeated after 1 week, 2 weeks, and 1 month after treatment with methylprednisolone 21-acetate (a derivative of methylprednisolone) 20 mg tablets (full dose 60 mg/day for 5 days and then tapering dose for another 10 days).

***Equipment***

These are the following:

- Sound-treated room: Transacoustic model no. RE241
- Pure tone audiometry: Amplivox 270 clinical audiometer
- Immittancemetry: Interacoustic AT235h

The duration of the study will range from 3 to 6 months. The protocol of the study was approved by the ethical committee (34857/08/2020). Informed consent was obtained from all participants included.

***Inclusion criteria***

- Age 18 to 60 years
- Subjects diagnosed as COVID-19 positive with PCR
- No otological complaints of pre-viral infection
- Tympanometry tested was a bilateral type A tympanogram reflecting normal middle ear pressure at the time of testing.
- MRI with contrast on petrous bone and inner was normal.

***Exclusion criteria***

- Age below 18 years or above 60 years
- Audiological disease history or hearing complaints
- Subjects with sudden hearing loss but not diagnosed as COVID-19 by PCR

### Statistical methods

The data were tabulated and analyzed using version 22 of SPSS. The Fisher’s exact test was used to compare numerical and percentage-based categorical variables. Using the Shapiro-Wilk test, it was revealed that continuous data were not normally distributed. They were shown using the median and interquartile range. For comparison between the values at different studied time points, the Friedman test was applied. When it gave significant results, post hoc tests (Wilcoxon signed-rank tests) were used for pairwise comparison between each two-time point. Furthermore, a comparison between patients with or without improvement as regards the ages, the onset of SNHL, and the time of starting treatment was made by the Mann-Whitney *U*-test. A difference was considered significant when the *P*-value was < 0.05.

## Results

This study included 20 patients with an age range of 23–57 years, with a mean of 43.2 and a standard deviation ± 10.8. There were six females and 14 males. As regards the affected ear, the right ear was affected in 14 cases (70%), and the left ear was affected in 6 cases (30%). The onset of hearing loss post-COVID infection ranged from 1 to 3 months with a median (IQR) of 2.0 (1.5–2.5) months. Nine patients (45%) complained of vertigo with hearing loss, and eighteen patients (90%) complained of tinnitus noticed with the onset of hearing loss. Thirty percent of patients had general diseases, including hypertension and DM. As regards COVID infection details, seven (35%) patients were hospitalized, and only one patient was hospitalized in ICU for 5 days.

Hearing loss diagnosed by pure tone audiometry had many configurations. Low-frequency sensorineural hearing loss was the most common finding in nine cases (45%), high frequency in six cases (30%), and flat configuration in the remaining 5 cases (25%).

The patients in this study varied in the time of starting corticosteroids or generally seeking medical care. This ranged from 4 to 21 days with a median (IQR) of 14 (5–21) days. Also, a subjective sensation of improvement in the hearing was noticed in thirteen patients (65%), which was highly correlated with pure tone results documented in follow-up evaluations.

**Fig. 1:**
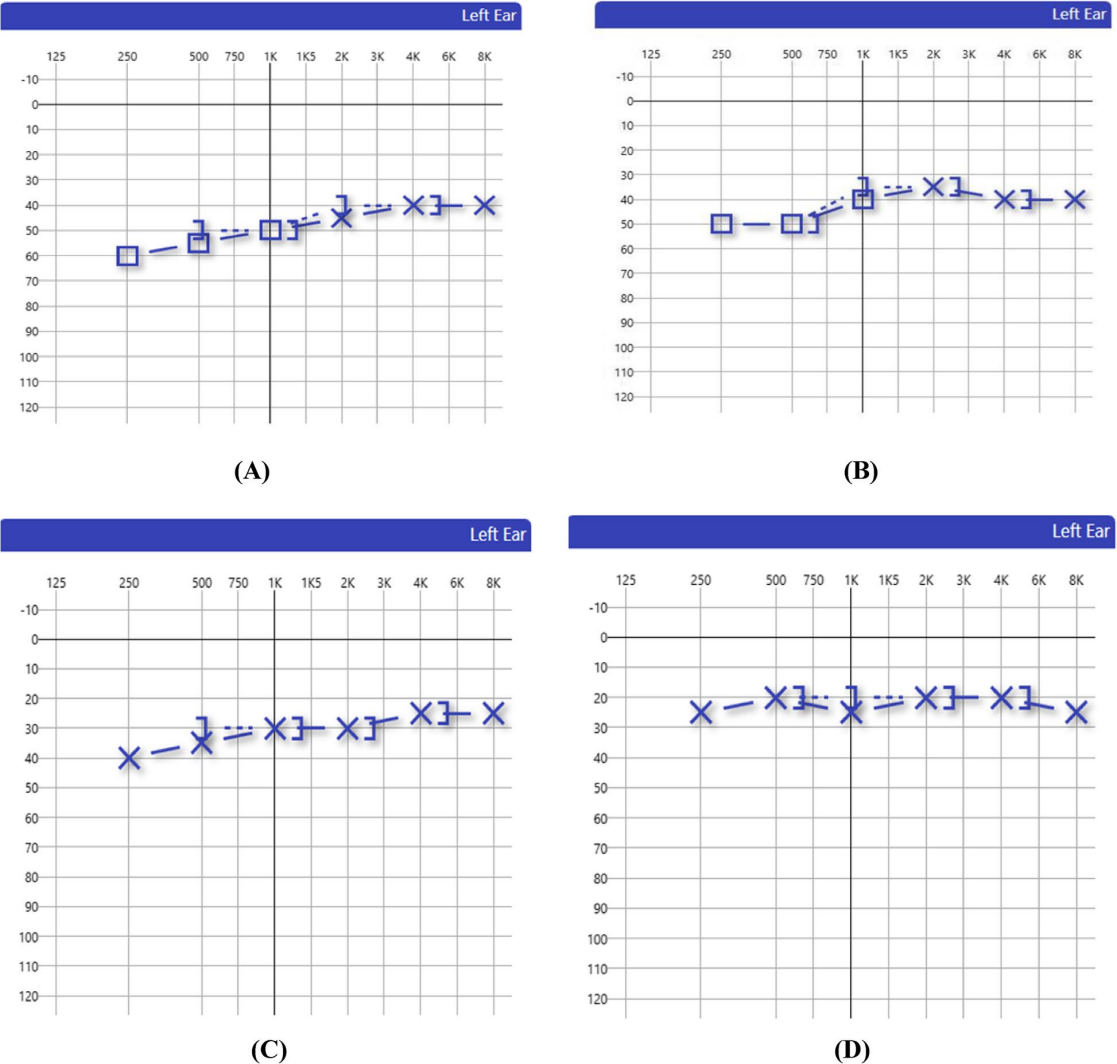
Follow up audiological evaluation of a male patient aged 37 years presented with left sudden low-frequency SNHL post COVID-19 infection. **A** Initial presentation PTA threshold. **B** PTA threshold after 1-week corticosteroid treatment. **C** PTA threshold after 2 weeks of corticosteroid treatment. **D** PTA threshold after 1 month of corticosteroid treatment

As regards the improvement recognized with the treatment course, thirteen patients (65%) of all twenty patients showed complete improvement 1 month after starting treatment, and seven patients (35%) showed no improvement even after 1 month. Also, a clear observation was recognized that the improvement is not complete except after 1 month, despite continuous improvement in consecutive testing and tapering of corticosteroid therapy Fig. 1.

Friedman test was used for comparison between the values of PTA and speech audiometry (SRT and SD%) at different studied time points (initial before starting the treatment, after 1 week, after 2 weeks, and after 1 month of treatment). This revealed a statistically significant difference in all tested frequencies (250–8000 Hz) and in SRT and SD%. When it gave significant results, post hoc test (Wilcoxon signed-rank tests) was used for pairwise comparison between each two-time point. This comparison revealed a statistically significant difference in all pairs, which indicates continuous improvement during follow-up evaluations (Tables 1 and 2).

**Table 1:** Comparison between the values of PTA at different studied time points (initial before starting of treatment, after 1 week, after 2 weeks, and after 1 month of treatment)

PTA frequency (Hz)	Evaluation	Median	Mean rank	Friedman test *p*-value	Post hoc test (Wilcoxon signed-rank test)
250 Hz	Initial	50.0	3.88	< 0.001*	P1 < 0.001* P2 < 0.001* P3 < 0.001* P4 < 0.001* P5 < 0.001* P6 = 0.001*
	One week	40.0	3.13		
	Two weeks	30.0	1.80		
	One month	25.0	1.20		
500 Hz	Initial	42.5	3.93	< 0.001*	P1 < 0.001* P2 < 0.001* P3 < 0.001* P4 < 0.001* P5 < 0.001* P6 = 0.010*
	One week	35.0	2.98		
	Two weeks	32.5	1.80		
	One month	27.5	1.30		
1000 Hz	Initial	42.5	3.90	< 0.001*	P1 < 0.001* P2 < 0.001* P3 < 0.001* P4 < 0.001* P5 < 0.001* P6 = 0.010*
	One week	35.0	2.95		
	Two weeks	30.0	1.83		
	One month	25.0	1.33		
2000 Hz	Initial	45.0	3.83	< 0.001*	P1 < 0.001* P2 < 0.001* P3 < 0.001* P4 < 0.001* P5 < 0.001* P6 = 0.014*
	One week	37.5	2.98		
	Two weeks	30.0	1.88		
	One month	30.0	1.33		
4000 Hz	Initial	45.0	3.80	< 0.001*	P1 = 0.001* P2 < 0.001* P3 < 0.001* P4 = 0.001* P5 < 0.001* P6 = 0.001*
	One week	45.0	2.85		
	Two weeks	35.0	2.05		
	One month	30.0	1.30		
8000 Hz	Initial	45.0	3.83	< 0.001*	P1 = 0.001* P2 < 0.001* P3 < 0.001* P4 < 0.001* P5 < 0.001* P6 = 0.005*
	One week	45.0	3.18		
	Two weeks	40.0	1.70		
	One month	35.0	1.30		

*Significant at *p* < 0.05. Pairwise comparison revealed significant differences (*P* < 0.05*). P1, initial versus 1 week. P2, initial versus 2 weeks. P3, initial versus 1 month. P4, 1 week versus 2 weeks. P5, 1 week versus 1 month. P6, 2 weeks versus 1 month

**Table 2:** Comparison between the values of speech audiometry (SRT and SD%) at different studied time points (initial before starting of treatment, after 1 week, after 2 weeks, and after 1 month of treatment) showing median and mean rank

Speech audiometry	Evaluation	Median	Mean rank	Friedman test *p*-value	Post hoc test (Wilcoxon signed-rank test)
Speech discrimination (SD%)	Initial	84.0	1.13	< 0.001*	P1 < 0.001* P2 < 0.001* P3 < 0.001* P4 = 0.001* P5 < 0.001* P6 = 0.002*
	One week	84.0	2.18		
	Two weeks	88.0	3.03		
	One month	96.0	3.68		
SRT (dB nHL)	Initial	45.0	4.0	< 0.001*	P1 < 0.001* P2 < 0.001* P3 < 0.001* P4 < 0.001* P5 < 0.001* P6 = 0.007*
	One week	35.0	2.88		
	Two weeks	35.0	1.83		
	One month	25.0	1.30		

*Significant at *p* < 0.05. Pairwise comparison revealed significant differences (*P* < 0.05*). P1, initial versus 1 week. P2, initial versus 2 weeks. P3, initial versus 1 month. P4, 1 week versus 2 weeks. P5, 1 week versus 1 month. P6, 2 weeks versus 1 month

Association between different factors and complete improvement was tested using the Mann-Whitney *U*-test. These prognostic factors include age, the onset of hearing loss, general diseases, time of starting steroids, and hearing loss configuration. Mann-Whitney and Fisher exact tests showed a significant association between hearing improvement and age, onset of hearing loss, and time of starting corticosteroid therapy (Table 3).

**Table 3:** Association between different factors and hearing improvement in patients in this study

			Hearing improvement		Mann-Whitney and Fisher exact tests
			No 7 (35.0%)	Yes 13 (65.0%)	*p*-value
Age (years)	Median (IQR)		51.0 (51.0–53.0)	36.0 (35.0–44.0)	0.011*
	Mean rank		15.0	8.08	
Sex	Female	N	1	5	0.354
		%	14.3%	38.5%	
	Male	N	6	8	
		%	85.7%	61.5%	
Onset of HL (months)	Median (IQR)		3.0 (2.0–3.0)	1.5 (1.5–2.0)	0.006*
	Mean rank		15.29	7.92	
Time of starting steroids (days)	Median (IQR)		21.0 (14.0–21.0)	7.0 (5.0–14.0)	0.008*
	Mean rank		15.14	8.0	
General disease	No	N	3	11	0.078
		%	42.9%	84.6%	
	Yes	N	4	2	
		%	57.1%	15.4%	
HL configuration	Flat	N	1	4	0.716
		%	14.3%	30.8%	
	High	N	3	3	
		%	42.9%	23.1%	
	Low	N	3	6	
		%	42.9%	46.2%	

*Significant at *P* < 0.05

## Discussion

Current research in otolaryngology focuses on the optimal route of steroid delivery for treating sensorineural hearing loss. To date, however, SSNHL in the context of COVID-19 has been extensively recognized, since COVID-19 has been identified in a number of recent case reports as the cause of SSNHL [15–18].

Despite the many symptoms linked with the SARS-CoV-2 virus, the relationship between COVID-19 and hearing has received little attention. Mustafa (2020) examined the transient-evoked optoacoustic emissions (TEOAE) of twenty SARS-CoV-2-positive patients and found that their pure high-frequency tone audiometry thresholds and TEOAE amplitudes were considerably lower than those of healthy individuals. This suggests that COVID-19 might be associated with cochlear injury [19].

The loss of hair cells and supporting cells of the organ of Corti in the cochlea of SSNHL patients in the absence of inflammatory cell infiltration suggests that the pathophysiology of idiopathic SSNHL may involve cellular stress pathways [20]. With ACE-2 receptors, alveolar epithelial cells and pulmonary endothelium cells are sensitive to SARS-CoV-2 infection. Moreover, SARS-CoV-2 generates an inflammatory response and an increase in cytokines, such as tumor necrosis factor-α, interleukin-1, and interleukin-6 [21].

Another theory is that COVID-19 causes microvascular changes (thrombus or embolus) that result in ischemic injury to the inner ear or auditory centers [22]. Multiple outcomes, including arterial and venous thrombosis and multiorgan failure, have been linked to COVID-19 infection. This might be due to endothelins, which can damage the auditory center of the temporal lobe, the cochlear nerve, and/or cochlear tissues [23].

Kılıç et al. discovered that one in five patients with SSNHL had a positive COVID-19 PCR screening test, showing that COVID-19 and SSNHL are linked and that COVID-19 may increase the prevalence of SSNHL. They found that SSNHL might be an asymptomatic sign of COVID-19 or the only symptom. Knowledge of such a generic COVID-19 presentation is essential for preventing the spread of infection during the current pandemic by isolation and early delivery of COVID-19-specific medication. After administering 200 mg of oral hydroxychloroquine twice daily for 5 days, the COVID-19-positive patient in this study was completely cured with SSNHL [24].

Beckers et al. revealed significant evidence between COVID-19 with hearing loss. They proposed adding PCR testing into the diagnostic assessment of SSNHL patients during the present epidemic. According to the researchers, SSNHL may be caused by micro-thrombosis in endothelial cells of the cochlear or central auditory pathway [25].

Fidan et al. noticed an increase in the frequency of SSNHL during COVID-19. In addition, 39 of 68 SSNHL patients have positive nasopharyngeal swabs for SARS-CoV-2 (57.4%). In addition, they discovered that the neuroinvasion and autoimmune models describe COVID-19 and cranial neuropathy the best [26].

Despite this, Chari et al. found that the incidence of SSNHL did not vary between March and May during the pandemic and was also the same as the previous year, demonstrating that the COVID-19 virus does not increase the incidence of SSNHL [27]. In addition, Aslan and içek found that the incidence of SSNHL did not statistically change between the 1-year period before and after the pandemic. They offered two justifications for this. First, the pandemic may have suspended healthcare services for patients. The number of patients with SSNHL caused by a virus may have decreased as a result of current limitations and preventative measures [28].

In the current study, the onset of hearing loss post-COVID infection ranged from 1 to 3 months with a median (IQR) of 2.0 (1.5–2.5) months which is a delayed onset complaint. Beckers et al. observed that SSNHL may manifest during the terminal phase of disease since many patients did not recognize their hearing loss until they were discharged from the hospital or critical care unit [29].

Fidan et al. studied the difference between 2019 and 2020 years regarding the duration of SSNHL and asked for medical acclaim. They found that this duration was longer in the 2020 group than in the 2019 group (median = 2.3 days vs. 0.6 days, respectively) (*P* < 0.001) [26].

Koumpa et al. observed unilateral sudden hearing loss in an ICU patient being monitored for COVID-19, despite the patient’s assertion that hearing loss was not among his or her symptoms upon hospital admission [30].

Even though oral corticosteroids are often used for SSNHL, the evidence of their effectiveness is still equivocal. The physician may prescribe oral corticosteroids as first-line therapy for the patient in compliance with AAO-HNS criteria, notwithstanding the scarcity of evidence supporting their efficacy [31].

Corticosteroids used to treat influenza and other viruses may delay viral RNA clearance but do not reduce mortality, according to Russell et al. They advised against taking corticosteroids in such a situation [32]. In contrast, according to studies conducted at the University of Oxford by Mahase, steroids lowered mortality in ventilated patients and/or those receiving oxygen assistance but had no effect on mortality in those who did not need oxygen support. Bell’s palsy and SSNHL cannot be considered distinct symptoms/signs of COVID-19; hence, effective corticosteroids will be administered to these people [33].

The steroid dose and the effect of starting therapy at different times are also debatable. Westerlaken et al. compared the efficacy of pulse therapy of 300 mg intravenous (IV) methylprednisolone to that of standard oral prednisolone therapy for SSNHL [34]. Eftekharian and Amizadeh found similar results in their study [35]. In addition, the German Association of Scientific Medical Societies (AWMF) advocated high-dose intravenous steroid treatment (250 mg) [36].

However, Alexiou et al. and Egli Gallo et al. have shown that high-dose systemic steroid therapy is superior to conventional systemic corticosteroid therapy [37].

Several variables influence the possibility of SSNHL recovery. Age is recognized as the most constant factor with a negative effect since the recovery rate for senior patients is much lower [38–40]. This is in line with the findings of the present investigation, which revealed a negative association between recovery and age.

In this investigation, there was no correlation between the presence of tinnitus or vertigo and the improvement of SSNHL. Except that, vertigo was frequent in severe instances (those with a lower PTA threshold), and tinnitus was common even in moderate ones. Several studies indicate that vertigo is connected with a bad outcome [11, 38]. This outcome has not, however, been constant [39, 40]. Multiple studies have shown that tinnitus is a positive [41], negative [11], and neutral [42] prognostic indicator.

Those who contact a physician within a week have a greater chance of recovering from hearing loss than those who wait longer [38–40]. This is similar to the results of the current study, which revealed a negative correlation between recovery and the duration of SSNHL development after COVID-19 infection.

Patients with severe hearing loss were less likely to recover than those with moderate hearing loss [38, 43].

In this investigation, there was no correlation between hearing recovery and hearing loss configuration. Chang et al. and Huy and Sauvageau investigated the configuration of hearing loss in individuals with moderate to severe hearing loss. They discovered that hearing recovery was decreased with a flat audiogram. For non-flat settings, an ascending audiogram exhibited a more favorable prognosis than a descending audiogram [11].

We found no correlation between hearing recovery and broad systemic disorders in our investigation. In some investigations, systemic comorbidities such as diabetes mellitus, hyperlipidemia, and/or hypertension were associated with a worse result [43].

Regarding the timing of treatment in relation to the development of symptoms, it has been traditional to start steroid medication as soon as possible (within the first 2 weeks). The Mann-Whitney and Fisher exact tests demonstrated a high connection between hearing improvement, and the date corticosteroid therapy was started in the current study. Numerous studies demonstrate that the length of time between the onset of symptoms and the initiation of therapy is detrimental to prognosis [38]. The recovery rates of patients who sought medical counsel and began treatment during the first week of sickness onset, within 2 weeks and 3 months later, were 87%, 52%, and 10%, respectively [39, 40, 44]. The AAO-HNS suggests beginning steroid treatment within 2 weeks [31].

## Conclusions

SSNHL has been widely recognized in the context of COVID-19 to date. Early corticosteroid therapy could help in the recovery of hearing, especially if the beginning of therapy was early in the first 2 weeks.

### Recommendations

The precise relationship between COVID-19 and neurological issues has not been established. Many studies are needed to understand this connection. The effectiveness of corticosteroids with different routes of administration (systemic, intratympanic, or combination) needs to be studied. Vestibular evaluation can also be examined in these patients, even with the absence of vertigo in the symptomatology.

## Data Availability

Data is available (if needed) for revision.
